# A simple nomogram for predicting infectious diseases in adult kidney transplantation recipients

**DOI:** 10.3389/fpubh.2022.944137

**Published:** 2022-08-31

**Authors:** Ruo-Yang Chen, Sheng Zhang, Shao-Yong Zhuang, Da-Wei Li, Ming Zhang, Cheng Zhu, Yue-Tian Yu, Xiao-Dong Yuan

**Affiliations:** ^1^Department of Urology, Renji Hospital, School of Medicine, Shanghai Jiaotong University, Shanghai, China; ^2^Department of Critical Care Medicine, Ruijin Hospital, School of Medicine, Shanghai Jiao Tong University, Shanghai, China; ^3^Department of Disease Prevention and Control, Ruijin Hospital, School of Medicine, Shanghai Jiao Tong University, Shanghai, China; ^4^Department of Critical Care Medicine, Renji Hospital, School of Medicine, Shanghai Jiao Tong University, Shanghai, China

**Keywords:** kidney transplantation, solid organ transplantation, nomogram, prediction model, infectious disease

## Abstract

**Objective:**

To investigate the risk factors of infectious diseases in adult kidney transplantation recipients and to establish a simple and novel nomogram to guide the prophylactic antimicrobial therapy.

**Methods:**

Patients who received kidney transplantation between January 2018 and October 2021 were included in the study and were divided into a training and a testing set at a 1:1 ratio. Risk factors correlated to infectious diseases were selected using a Least Absolute Shrinkage and Selection Operator (LASSO) regression model. The prediction model was built by incorporating the variables selected by the LASSO model into a logistic regression equation. Calibration curves and receiver operating characteristic (ROC) curves were also applied to assess the model calibration and discrimination. A nomogram consisting of the selected factors was established to provide individualized risks of developing infections. Decision curve analysis (DCA) was adopted to estimate the net benefit and reduction in interventions for a range of clinically reasonable risk thresholds.

**Results:**

In all, 863 adult kidney recipients were included in the study, and 407 (47.16%) of them developed infectious diseases during the 3-year follow–up period. A total of 8 variables were selected using LASSO regression and were retained for subsequent model construction and infection prediction. The area under the curve (AUC) was 0.83 and 0.81 in the training and testing sets, with high F scores of 0.76 and 0.77, sensitivity of 0.76 and 0.81, and specificity of 0.88 and 0.74, respectively. A novel nomogram was developed based on 8 selected predictors (requirement for albumin infusion, requirement for red blood cell infusion, triglyceride, uric acid, creatinine, globulin, neutrophil percentage, and white blood cells). The net benefit indicated that the nomogram would reduce unnecessary interventions at a wide range of threshold probabilities in both sets.

**Conclusions:**

Adult kidney transplantation recipients are high-risk hosts for infectious diseases. The novel nomogram consisting of 8 factors reveals good predictive performance and may promote the reasonable antimicrobial prescription. More external validations are required to confirm its effectiveness for further clinical application.

## Introduction

Along with the ongoing advances of modern science, an increasing number of solid organ transplantations (SOTs) have been performed in the last decade, and the life quality of those with chronic organ failure has been greatly improved (1). However, due to the application of glucocorticoids and immunosuppressive agents, secondary infection in transplant recipients is an unavoidable problem and has become the major cause of mortality (2). Therefore, great efforts have been made to manage the infectious diseases appropriately in these immunocompromised hosts (ICHs). These strategies include establishment of transplant infectious diseases (TID) subspecialty, development of relevant practice guidelines, and setting up epidemiological database (3, 4).

Antimicrobial prophylaxis was once considered an effective strategy to prevent the infectious complications in SOT recipients. However, the complexity of the immune function and the atypical clinical manifestations make it difficult to achieve precise treatment (5). Remaining questions include when prophylactic antimicrobial treatment should be started, and who can benefit the most from such therapy are still in debate. Moreover, inappropriate use of antibiotics to avoid life-threatening infections is not uncommon, and it may lead to undesirable consequences, including antibiotic resistance and adverse drug effects (ADEs) (6).

Some prediction models or algorithms based on artificial intelligence and machine learning have been established to assist decision-making and assess the prognosis of infectious diseases (7, 8). However, few simple and reliable prediction model about the infections of SOT recipients is available (9, 10).

More than 1,000 kidney transplant operations have been performed in Renji hospital in the last 3 years and nearly half of them had infectious diseases during the follow-up period. The clinical characteristics, laboratory test results and state of illness are properly documented in our hospital information system (HIS). We also find that variables of the recipients with or without infectious diseases are quite different. Therefore, we hypothesized that the risk factors for infections can be identified from the clinical information of these kidney transplantation recipients. Thus, a comprehensive classification system based on the selected factors can be developed and a novel nomogram may also be established by incorporating such factors. It is anticipated that recipients with infectious diseases can be recognized quickly by the nomogram and evidence-based use of prophylactic antibiotics can also be promoted.

## Materials and methods

### Summary of study methodology

This study was conducted in Renji Hospital, which is affiliated with Shanghai Jiaotong University School of Medicine. The hospital is one of the largest organ transplant centers in China and the number of kidney transplantation is about 400 per year. Information on kidney transplantation recipients between January 2018 and October 2021 were retrieved from the HIS by four attending physicians (R-Y C, S Z, S-Y Z and D-W Li). The design and implementation of the study carefully followed the Transparent Reporting of a multivariable prediction model for Individual Prognosis Or Diagnosis (TRIPOD) statement (11). The Ethics Committee of Renji Hospital provided an exemption from the requirement for informed consent, since only previously clinical data were used, and the privacy of the patients was protected. For each patient enrolled, an identification number was issued to ensure anonymity.

### Study population, sample size, and disease definition

Patients aged between 18 and 80 years old who had received a kidney transplantation and were followed up regularly were included in the study. Those who met any of the following criteria were excluded: (I) had a chronic infectious disease; (II) urinary tract infection had been diagnosed in the donor; and (III) incomplete follow-up data. Thus, none of the recipients was prescribed antibiotics before transplantation.

All the patients included were divided into a training set and a testing set at a ratio of 1:1. Logistic regression model was applied for nomogram construction in our study. Based on the Events Per Variable (EPV) criterion and the sample size guideline for logistic regression from observational studies, a minimum sample size of 500 patients was recommend (12). More than one thousand adult kidney transplantation recipients were regular follow up during the study period and the sample size could fulfill the requirement.

Antibiotic prophylaxis is one of the most important components of our perioperative management process. Third generation cephalosporins were often prescribed to those with living related kidney transplantation for about 1 week. Those with allograft renal transplantation were often prescribed carbapenems during their hospital stay. Infectious diseases in kidney transplantation recipients included pneumonia, urinary tract infection, bacteremia, abdominal infection, central nervous system infection and skin and soft tissue infection. The definitions of these infectious diseases followed the diagnostic criteria of the European Society of Clinical Microbiology and Infectious Diseases (ESCMID) carefully (13). Pathogenic bacteria were isolated using cultures in the microbiological laboratory, and pathogenic viruses were identified using polymerase chain reaction (PCR). Recipients with hypoalbuminemia (defined as the serum albumin level was <35 g/L) received an albumin infusion before the operation (14), and those with renal anemia or hemoglobin levels lower than 70 g/L received a blood infusion (15, 16).

### Clinical data collection and assessment

Data about the recipients was retrieved from the HIS and included the following aspects: (I) information on infectious diseases of the donors and kidneys; (II) information on infectious diseases of the kidney transplantation recipients; (III) clinical characteristics of the kidney transplantation recipients, including gender, age, comorbidity (chronic renal dysfunction, hypertension, and diabetes); (IV) antirejection medications prescription, including glucocorticoid, anti-proliferation, and immunosuppressive agents; and (V) laboratory tests of blood samples of the recipients during the follow-up period. The average follow-up period for the first 90 days after transplantation was once a week and then every other week over the next 180 days. Data from the last follow-up before infection was included in the study.

### Statistical analysis

Data relating to continuous variables were expressed as median [interquartile range (IQR)] or mean ± standard deviation (SD). For categorical variables, percentages were calculated and Chi square tests were performed. Normally distributed continuous variables were compared using the Student's *t*-test, whereas non-normally distributed variables were compared using the Wilcoxon rank sum test.

To identify the risk factors for infectious diseases in kidney transplantation recipients, the Least Absolute Shrinkage and Selection Operator (LASSO) regression model with the “lambda.min” criterion was applied for variable selection. Lasso algorithm allowed variables selection by forcing the coefficients of non-significant variables to shrink to zero through a penalty. In this process, potential confounding variables would be removed and only significant independent variables would be retained for outcome prediction (occurrence of infectious disease or not). It excluded non-essential variables and retained a subset of the most important variables for outcome prediction (occurrence of infectious disease or not). The prediction model was built by incorporating the variables selected by the LASSO model into a logistic regression equation. Model discrimination was assessed using receiver operating characteristic (ROC) curves, which included sensitivity, specificity, correctly classified rate, positive likelihood ratio, and negative likelihood ratio. A calibration curve to assess the goodness-of-fit, accuracy, and applicability of the predictive nomogram in the training and testing sets was generated. To reduce the risk of overfitting, the whole cohort was randomly divided into the training and testing sets at a ratio of 1:1. They were used for model development and unbiased assessment of model performance, respectively. Finally, a novel nomogram consisting of the selected predictors was established to provide an individualized risk of the infectious disease occurrence. Decision curve analysis (DCA) was adopted to evaluate the net benefit for the prediction model in comparison to default strategies, which assume that all or no observations received interventions (17, 18). We also converted the net benefit into the net reduction in interventions to show the reduction in the number of unnecessary interventions per 100 patients based on the prediction model. To achieve this, the graphs of net benefit and the net reduction in interventions were plotted against a range of clinically reasonable threshold probabilities for the training and testing sets.

Statistical analyses were performed using R (University of Auckland, New Zealand, Version 3.6.2) and SPSS 21.0 (IBM Corp., Armonk, NY, USA). A two-sided *P*-value < 0.05 was considered statistically significant.

## Results

### Clinical features of the study population

**Figure 1:**
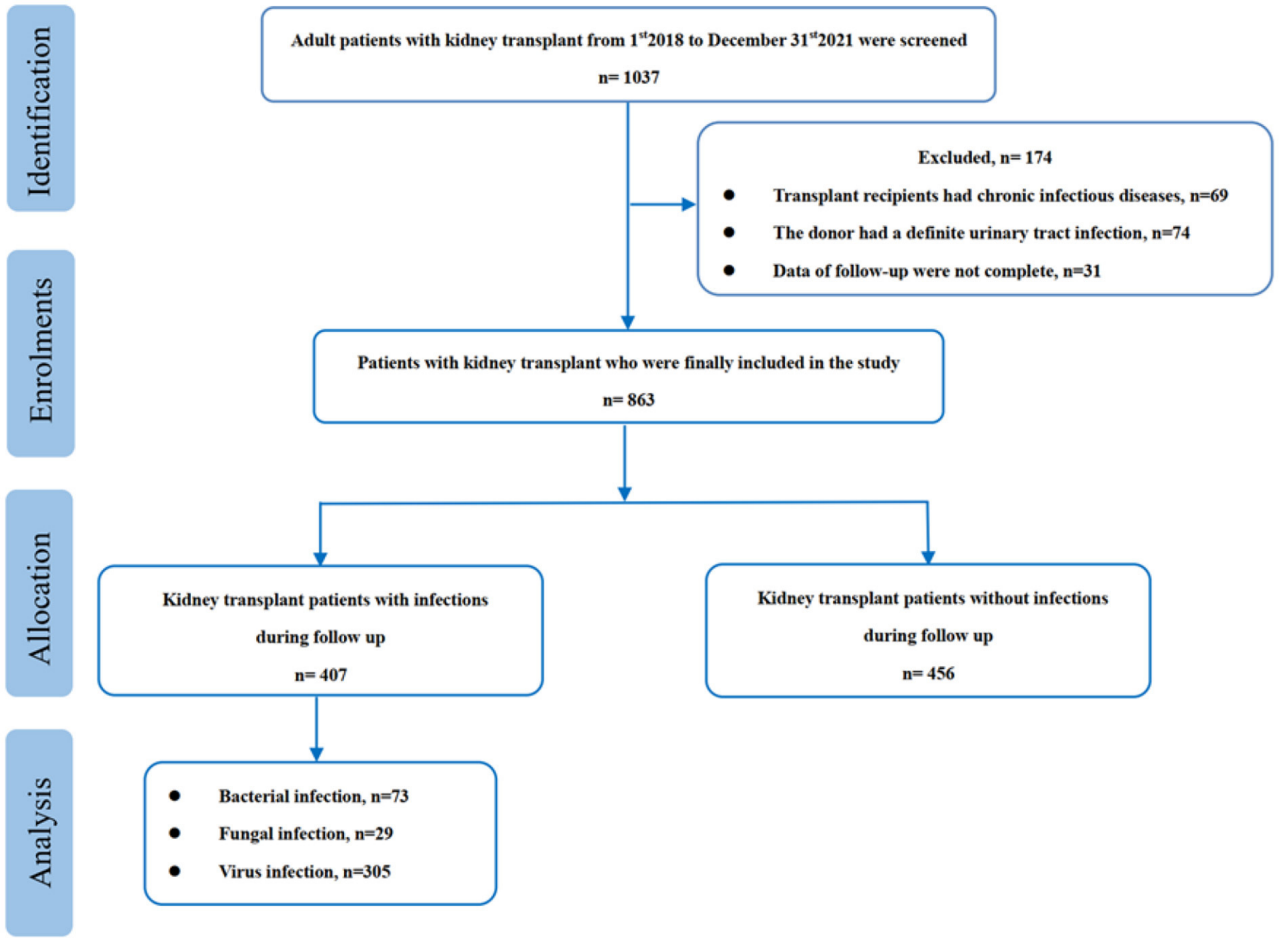
Flow chart of the study.

During the study period, 1,037 adult kidney transplantation recipients were initial screened and 863 of them were finally enrolled in our study. Among them, 407 (47.2%) had infectious diseases during the follow-up period, and most of them were viral infections (305, 74.9%) (Figure 1). The pathogenic viruses were identified by PCR and the majority of them were cytomegalovirus (CMV) and respiratory syncytial virus (RSV). No patient with severe acute respiratory syndrome coronavirus 2 (SARS-CoV-2) infection was detected. Among the 73 bacterial isolates, 7 were identified as Methicillin-resistant Staphylococcus aureus (MRSA), 29 were resistant to carbapenems and 33 were resistant to third generation cephalosporins.

The median time from discharge to infection occurrence was 62.3 (46.9, 90.6) days and the most common type of infection was pneumonia (289, 71.0%), followed by urinary tract infection (72, 17.7%). Moreover, 31 recipients had two or more sites infection. It revealed that most of the patients with infectious diseases needed red blood cell (RBC) transfusion (72.7%) and albumin (85.3%) infusion throughout the follow-up period due to anemia or hypoalbuminemia (Table 1).

**Table 1:** Demographics and clinical characteristics of patiovents with or without infectious disease.

**Variables**	**Total**	**Without** **infection**	**With** **infection**	***P*** **-value**
	**(*n =* 863)**	**(n = 456)**	**(n = 407)**	
Gender, *n* (%)				1.000
Female	344 (39.9)	182 (39.9)	162 (39.8)	
Male	519 (60.1)	274 (60.1)	245 (60.2)	
Age, median (IQR), years	43 (35, 52)	42 (35, 52)	43 (35, 52)	0.736
Blood type, *n* (%)				0.468
O type	301 (34.9)	155 (34.0)	146 (35.9)	
A type	255 (29.5)	128 (28.1)	127 (31.2)	
B type	208 (24.1)	116 (25.4)	92 (22.6)	
AB type	99 (11.5)	57 (12.5)	42 (10.3)	
Dialysis before operation, *n* (%)				0.315
No	45 (5.2)	20 (4.4)	25 (6.1)	
Yes	818 (94.8)	436 (95.6)	382 (93.9)	
Diabetes, *n* (%)				0.457
No	846 (98.1)	445 (97.6)	401 (98.5)	
Yes	17 (1.9)	11 (2.4)	6 (1.5)	
Hypertension, *n* (%)				0.590
No	720 (83.4)	377 (82.7)	343 (84.3)	
Yes	143 (16.6)	79 (17.3)	64 (15.7)	
Delayed graft function, *n* (%)				<0.001
No	664 (76.9)	377 (82.7)	287 (70.5)	
Yes	199 (23.1)	79 (17.3)	120 (29.5)	
Glucocorticoid, *n* (%)				0.384
Methylprednisolone	773 (89.6)	403 (88.4%)	370 (90.9%)	
Metacortandracin	78 (9.0)	47 (10.3%)	31 (7.62%)	
Prednisolone	12 (1.4)	6 (1.32%)	6 (1.47%)	
Tacrolimus, *n* (%)				0.329
No	124 (14.4)	60 (13.2)	64 (15.7)	
Yes	739 (85.6)	396 (86.88)	343 (84.3)	
Immunity induction, *n* (%)				0.577
Thymoglobulin	820 (95.0)	431 (94.5)	389 (95.6)	
Basiliximab	43 (5.0)	25 (5.5)	18 (4.4)	
WBC, median (IQR), 10^9^/L	7.1 (5.6, 9.3)	7.8 (6.1,10.3)	6.7 (5.0, 8.2)	<0.001
Neutrophil percentage, median (IQR), %	80.3 (74.0, 85.8)	82.2 (77.3, 86.9)	78.0 (70.0, 82.7)	<0.001
Platelet, median (IQR), 10^9^/L	215 (168, 264)	216 (173, 268)	215 (164, 254)	0.058
Globulin, median (IQR), g/L	22.7 (20.2, 25.4)	23.5 (21.3, 26.9)	22.5 (19.1, 23.2)	<0.001
Albumin infusion:				<0.001
No	261 (30.2)	201 (44.1)	60 (14.7)	
Yes	602 (69.8)	255 (55.9)	347 (85.3)	
RBC infusion:				<0.001
No	361 (41.8)	250 (54.8)	111 (27.3)	
Yes	502 (58.2)	206 (45.2)	296 (72.7)	
Prealbumin, median (IQR), mg/L	291 (71.1)	283 (66.2)	299 (75.2)	0.001
Alanine transaminase, median (IQR), U/L	16.0 (11.0, 25.5)	17.0 (11.0, 29.0)	16.0 (11.0, 21.0)	0.005
Aspartate aminotransferase, median (IQR), U/L	15.0 (12.0, 19.0)	15.0 (12.0;19.0)	15.0 (13.0, 19.0)	0.307
Direct bilirubin, median (IQR), umol/L	2.6 (2.0, 3.5)	2.6 (2.1, 3.8)	2.6 (2.0, 3.1)	0.004
Total bilirubin, median (IQR), umol/L	7.7 (6.3, 10.0)	7.7 (6.4, 10.6)	7.7 (6.2, 9.3)	0.090
Urea, median (IQR), mmol/L	9.6 (7.6, 13.5)	9.5 (7.2, 12.7)	9.6 (7.9, 14.2)	0.003
Creatinine, median (IQR), umol/L	117 (93, 162)	107 (82, 136)	128 (108, 217)	<0.001
Uric acid, median (IQR), umol/L	304 (252, 370)	282 (230, 344)	321 (288, 394)	<0.001
Triglyceride, median (IQR), mmol/L	1.8 (1.6, 2.1)	1.8 (1.5, 2.2)	1.8 (1.7, 2.1)	0.474
Cholesterol, median (IQR), mmol/L	4.5 (4.1, 4.9)	4.5 (3.8, 4.6)	4.5 (4.5, 5.2)	<0.001
Blood glucose, median (IQR), mmol/L	4.9 (4.5, 5.4)	4.9 (4.3, 5.2)	4.9 (4.9, 5.6)	<0.001

### Predictive factors identification and validation

**Figure 2:**
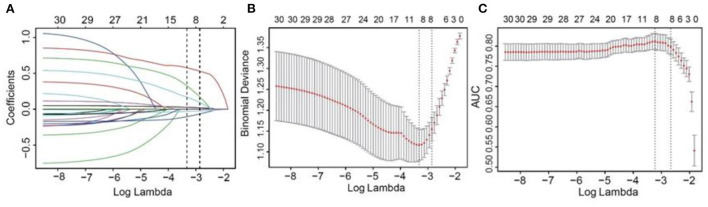
Selection of risk factors of infectious diseases using LASSO regression algorithm. A vertical line was plotted at the given lambda, selected by 10-fold cross-validation with minimum classification error and minimum classification error plus one standard error. For the optimal lambda that gives minimum classification error, 8 features with a non-0 coefficient were selected. **(A)** LASSO coefficient profiles of the candidate variables. **(B)** The binomial deviance with 95% CI (y-axis) was plotted against log (lambda) (bottom x-axis), when the number of included variables were changed (upper x-axis). **(C)** The AUCs with 95% CI (y-axis) were plotted against log (lambda) (bottom x-axis), when the number of included variables were changed (upper x-axis). LASSO, the Least Absolute Shrinkage and Selection Operator; CI, confidence interval; AUC, areas under the curve.

The 863 patients included were randomly divided into a training and a testing set at a 1:1 ratio. Basic clinical data of kidney transplantation recipients in the training and testing sets are shown in Table 2, and most of the variables included were evenly distributed. A LASSO model with the “lambda.min” criterion was applied to select variables. Thirty variables were screened initially, and 8 of them were retained for subsequent prediction model construction (Figure 2).

**Table 2:** Demographics and clinical characteristics of patients in testing and training groups.

**Variables**	**Total**	**Testing** **group**	**Training** **group**	***P*** **-value**
	**(*n =* 863)**	**(*n =* 431)**	**(*n =* 432)**	
Gender, *n* (%)				0.461
Female	344 (39.9)	166 (38.5)	178 (41.2)	
Male	519 (60.1)	265 (61.5)	254 (58.8)	
Age, median (IQR), years	43 (35, 52)	42 (35, 52)	43 (35, 52)	0.615
Infection, *n* (%)				0.261
No	456 (52.8)	219 (50.8)	237 (54.9)	
Yes	407 (47.2)	212 (49.2)	195 (45.1)	
Infection types, *n* (%)				0.337
Non-infection	456 (52.8)	219 (50.8)	237 (54.9)	
Bacterial infection	73 (8.5)	34 (7.9)	39 (9.0)	
Fungal infection	29 (3.4)	13 (3.0)	16 (3.7)	
Virus infection	305 (35.3)	165 (38.3)	140 (32.4)	
Blood type, *n* (%)				0.126
O type	301 (34.9)	151 (35)	150 (34.7)	
A type	255 (29.5)	136 (31.6)	119 (27.5)	
B type	208 (24.1)	90 (20.9)	118 (27.3)	
AB type	99 (11.5)	54 (12.5)	45 (10.4)	
Dialysis before operation, *n* (%)				0.766
No	45 (5.2)	21 (4.9)	24 (5.6)	
Yes	818 (94.8)	410 (95.1)	408 (94.4)	
Diabetes, *n* (%)				0.621
No	846 (98.0)	421 (97.7)	425 (98.4)	
Yes	17 (2.0)	10 (2.3)	7 (1.6)	
Hypertension, *n* (%)				0.572
No	720 (83.4)	356 (82.6)	364 (84.3)	
Yes	143 (16.6)	75 (17.4)	68 (15.7)	
Outcomes, *n* (%)				0.551
Survival	852 (98.7)	427 (99.1)	425 (98.4)	
Non-survival	11 (1.3)	4 (0.9)	7 (1.6)	
Delayed graft function, *n* (%)				0.323
No	664 (76.9)	325 (75.4)	339 (78.5)	
Yes	199 (23.1)	106 (24.6)	93 (21.5)	
Glucocorticoid, *n* (%)				0.485
Methylprednisolone	773 (89.6)	383 (88.9)	390 (90.3)	
Metacortandracin	78 (9)	40 (9.3)	38 (8.8)	
Prednisolone	12 (1.4)	8 (1.9)	4 (0.9)	
Anti-proliferation, *n* (%)				0.281
Mycophenolate mofetil	740 (85.7)	373 (86.5)	367 (85.0)	
Mycophenol sodium	123 (14.3)	58 (13.5)	65 (15.0)	
Tacrolimus, *n* (%)				0.488
No	124 (14.4)	66 (15.3)	58 (13.4)	
Yes	739 (85.6)	365 (84.7)	374 (86.6)	
Immunity induction, *n* (%)				0.748
Thymoglobulin	820 (95)	408 (94.7)	412 (95.4)	
Basiliximab	43 (5)	23 (5.3)	20 (4.6)	
WBC, median (IQR), 10^9^/L	7.1 (5.6, 9.3)	7.1 (5.4, 9.4)	7.1 (5.6, 9.2)	0.489
RBC, median (IQR), 10^12^/L	3.1 (2.6, 3.7)	3.1 (2.6, 3.7)	3.1 (2.6, 3.6)	0.263
Neutrophil percentage, median (IQR), %	80.3 (74, 85.8)	80.3 (73.4, 86)	80.3 (74.4, 85.6)	0.680
Lymphocyte percentage, median (IQR), %	11.1 (6.6, 17.3)	11.1 (6, 17.9)	11.1 (7.2, 17)	0.182
Hemoglobin, median (IQR), g/L	92 (80, 108)	92 (80, 109)	92 (80, 105.2)	0.363
Platelet, median (IQR), 10^9^/L	215 (168.5, 264)	215 (171, 264.5)	215 (164, 263.2)	0.485
Globulin, median (IQR), g/L	22.7 (20.2, 25.4)	22.7 (20.1, 25.5)	22.7 (20.2, 25.3)	0.926
Albumin, median (IQR), g/L	37.3 (34.3, 41.6)	37.3 (34.3, 41.9)	37.3 (34.3, 41.3)	0.263
Prealbumin, median (IQR), mg/L	286 (248, 328.5)	286 (249, 321.5)	286 (245.8, 333)	0.704
Alanine transaminase, median (IQR), U/L	16 (11, 25.5)	16 (12, 26)	16 (11, 25)	0.602
Aspartate aminotransferase, median (IQR), U/L	15 (12, 19)	15 (12, 19)	15 (12, 19)	0.846
Direct bilirubin, median (IQR), umol/L	2.6 (2, 3.5)	2.6 (2, 3.5)	2.6 (2.1, 3.5)	0.173
Total bilirubin, median (IQR), umol/L	7.7 (6.3, 10)	7.7 (6, 10)	7.7 (6.4, 10.1)	0.146
Urea, median (IQR), mmol/L	9.6 (7.6, 13.5)	9.6 (7.7, 13.1)	9.6 (7.6, 13.8)	0.905
Creatinine, median (IQR), umol/L	117 (93, 161.5)	117 (95, 162)	117 (91, 159.2)	0.381
Uric acid, median (IQR), umol/L	303.5 (251.5, 370)	303.5 (254.5, 372.1)	303.5 (249.8, 367.2)	0.270
Triglyceride, median (IQR), mmol/L	1.8 (1.6, 2.1)	1.8 (1.6, 2.2)	1.8 (1.6, 2.1)	0.814
Cholesterol, median (IQR), mmol/L	4.5 (4.1, 4.9)	4.5 (4, 4.9)	4.5 (4.1, 4.9)	0.847
Blood glucose, median (IQR), mmol/L	4.9 (4.5, 5.4)	4.9 (4.6, 5.5)	4.9 (4.5, 5.4)	0.675
Albumin infusion:				0.982
No	261 (30.2)	131 (30.4)	130 (30.1)	
Yes	602 (69.8)	300 (69.6)	302 (69.9)	
RBC infusion:				0.805
No	361 (41.8)	178 (41.3)	183 (42.4)	
Yes	502 (58.2)	253 (58.7)	249 (57.6)	

**Figure 3:**
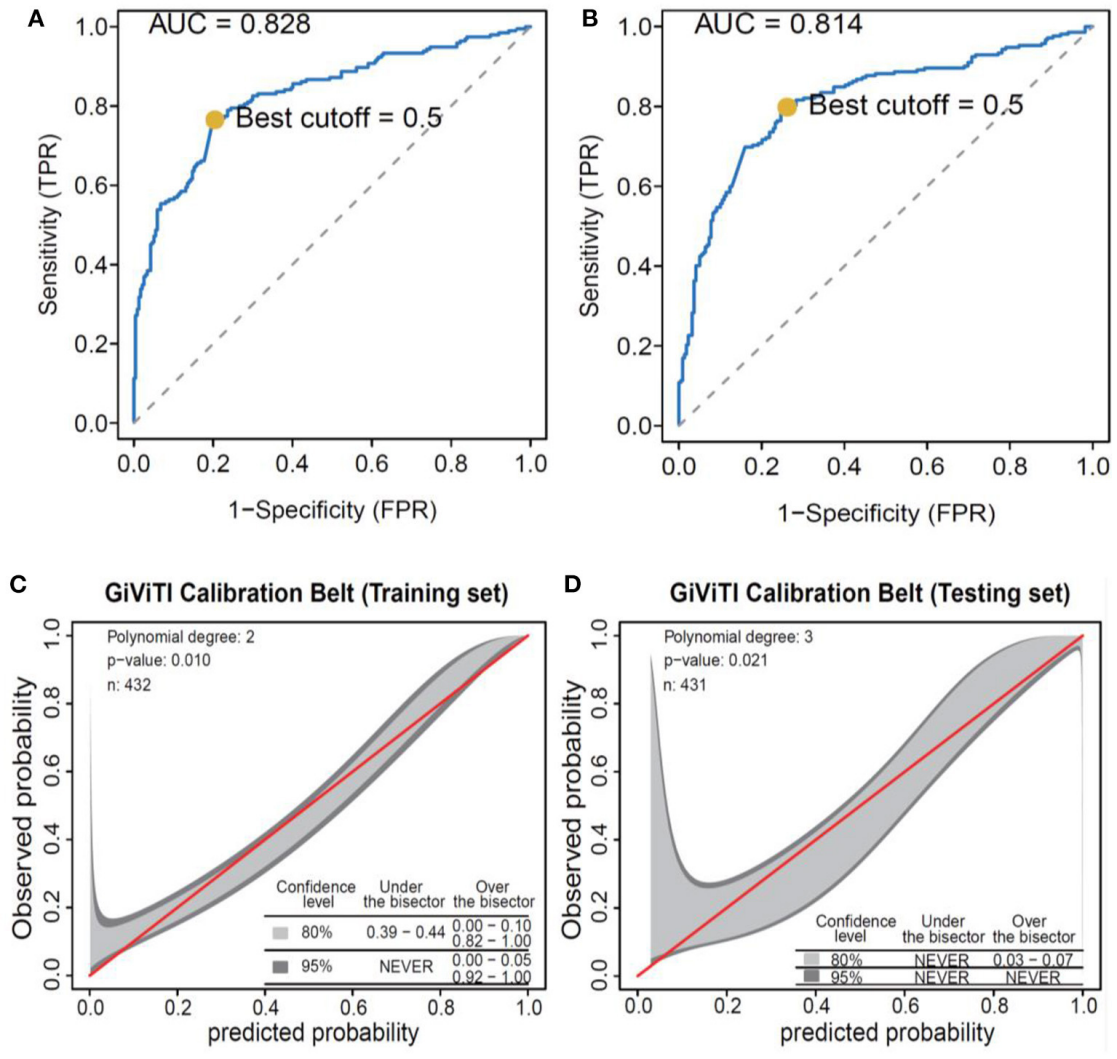
Performance of the logistic regression algorithm in infectious disease prediction. **(A)** ROC curves of the training set. **(B)** ROC curves of the testing set. **(C)** GiViTI calibration curves of the training set. **(D)** GiViTI calibration curves of the testing set. ROC, receiver operating characteristic.

The prediction model was built based on the 8 selected variables using a logistic regression equation. The parameters of the ROC curve at the optimal cut-off value according to different models were documented (Table 3; Figure 3). It was demonstrated that the area under the curve (AUC) was 0.83 and 0.81 in the training set and testing set, respectively, with high sensitivity (0.76 and 0.81, respectively), specificity (0.88 and 0.74, respectively), and F scores (0.76 and 0.77, respectively). Moreover, calibration curves revealed good agreements between predicted and observed probability for infectious diseases in both sets.

**Table 3:** Parameters of ROC curves for prediction of infectious diseases in training set and testing set.

**Data set**	**Cutoff**	**ACC**	**SENS**	**SPEC**	**PPV**	**NPV**	**pDLR**	**nDLR**	**FSCR**
Training set	0.50	0.78	0.76	0.80	0.76	0.81	3.85	0.29	0.76
Testing set	0.50	0.77	0.80	0.74	0.75	0.79	3.11	0.27	0.77

ACC, Overall accuracy of classification; SENS, Sensitivity; SPEC, Specificity; PPV, Positive predictive value; NPV, Positive predictive value; pDLR, Positive diagnostic likelihood ratio; nDLR, Negative diagnostic likelihood ratio; FSCR, F-score.

### Estimating the efficacy probability of infectious diseases using the nomogram

**Figure 4:**
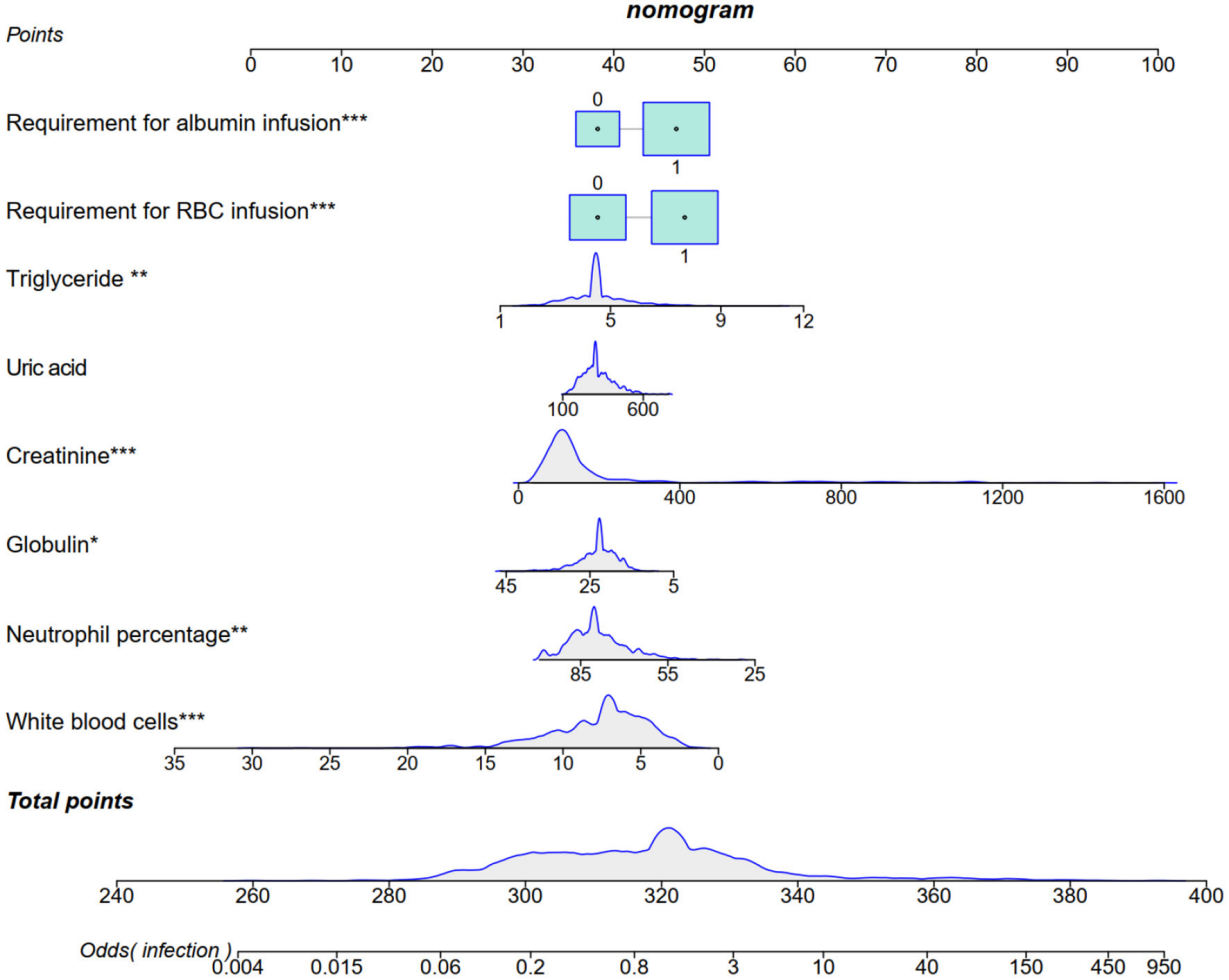
A nomogram to predict infectious diseases was developed using the predictors selected using LASSO. ^*^P < 0.05, ^**^P < 0.01, ^***^P < 0.001. LASSO, Least Absolute Shrinkage and Selection Operator.

We incorporated 8 selected predictors as prognostic features for the nomogram: requirements for albumin and RBC infusions, and levels of triglyceride, uric acid, creatinine, globulin, neutrophil percentage, and white blood cell (WBC). The nomogram can be utilized to predict individualized risk of infectious diseases in patients with kidney transplantation (Figure 4).

### Assessment of the nomogram performance based on the DCA

**Figure 5:**
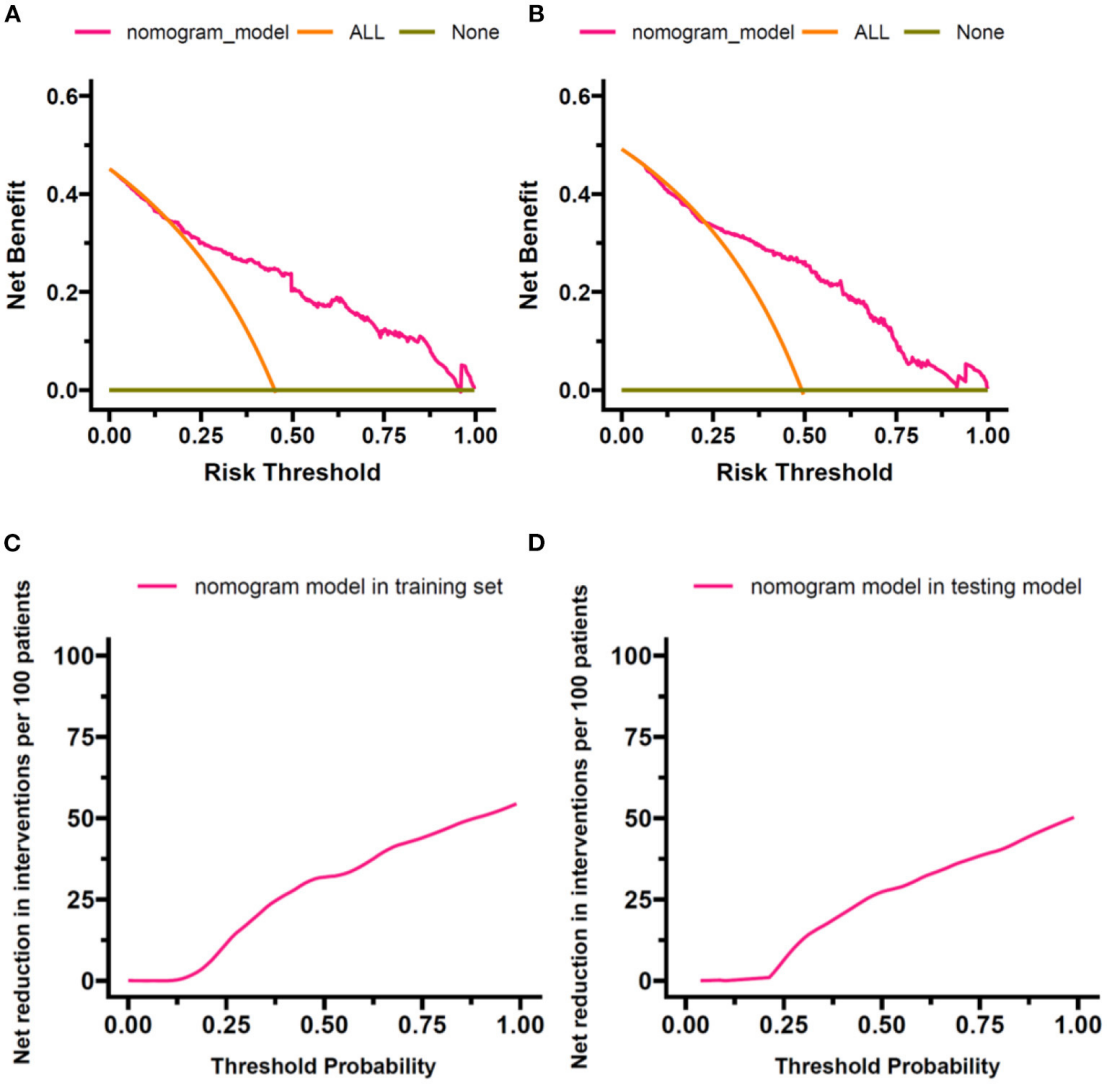
Results of DCA. DCA was performed to compare the nomogram-based decision with default strategies, which assume that all or no observations received interventions. **(A)** Net benefit against threshold probability in the training set. **(B)** Net benefit against threshold probability in the testing set. **(C)** Net reduction in interventions per 100 patients against threshold probability in the training set. **(D)** Net reduction in interventions per 100 patients against threshold probability in the testing set. DCA, decision curve analysis.

The DCA indicated the superior net benefit of the prediction model compared with default strategies, which assume that all or no observations received interventions in the training and testing sets (Figures 5A,B). The results of DCA were also demonstrated by converting the net benefit into the reduction in interventions per 100 patients. As shown in Figures 5C,D, a clinical strategy based on the nomogram would decrease the number of unnecessary interventions at a wide range of threshold probabilities in the training and testing sets.

## Discussion

A total of 863 recipients of kidney transplantations were enrolled in our study, and a novel nomogram consisting of 8 selected variables was established to predict the development of infectious diseases. Our study demonstrated that the requirements for albumin and RBC infusions, the levels of triglyceride, uric acid, creatinine, globulin, neutrophil percentage, and WBC should be carefully monitored during the follow-up period of the adult kidney transplantation recipients. It is indicated that the novel nomogram can improve the rational use of antibiotics and it may be helpful in decreasing the occurrence of antimicrobial resistance.

Kidney transplantation really improves the long-term outcomes in those with chronic renal failure, while opportunistic infection is still an enormous challenge. Thus, the recipients should be carefully evaluated for the risk of infection occurrence, and evidence-based prophylactic antibiotics prescription is recommended (19). Nearly half of the recipients enrolled in our study were infected during the 3-year follow-up period, and most of them were diagnosed with viral infections (74.9%), which included cytomegalovirus (CMV) and BK virus (20). The typical symptoms of viral infection are fever and fatigue. Above all, leukopenia and a low percentage of neutrophils can often be found from the complete blood count (CBC) (21). Therefore, it is reasonable that the count of WBC and the percentage of neutrophils in kidney recipients with infectious diseases are lower than those without infections.

Serum globulin level is one of the most important factors which can reflect the status of humoral immunity. The multiple roles of globulin in the host defense include regulation of the immune system, pathogen clearance, mucosal immunity, and toxin neutralization (22). Not surprisingly, the presence of posttransplant hypogammaglobulinemia is known as an independent risk factor that may induce infectious diseases. Our study shows that the level of serum globulin is lower in the infected than the non-infected group, which is in line with a meta-analysis that included 18 studies (23).

Graft-versus-host disease (GVHD) is another major problem for kidney transplantation, which may present as high levels of uric acid, creatinine and renal vascular resistance (24). Thus, glucocorticoids and immunosuppressors should be prescribed, which may lead to further impairment of immune function and a higher probability of opportunistic infections. Nutritional status is another important indicator that cannot be neglected in assessing the risk of infection (25). It has already been demonstrated that a low concentration of serum albumin is strongly associated with reduced kidney function, which may also cause infection in kidney recipients (26). Thus, albumin infusion is needed for those with a low level of serum albumin during the follow-up period, which has been recognized as an independent risk factor for infection. Hemoglobin is a protein with multiple functions. It is not only responsible for carrying oxygen in an organism but also has implication for the genetic resistance to infection (27). The hemoglobin peptide library can produce different biological effects, which include antimicrobial hemoglobin-derived peptides. Then, the antimicrobial hemoglobin-derived peptides can produce antibacterial effects, thereby reducing inflammation caused by microbial infections (28, 29). Therefore, the requirement for RBC infusion was also included in our nomogram as a risk factor for infection.

Lipids are indispensable in the infection process, although they are usually associated with the metabolic and nutritional status of patients (30). In the present study, triglyceride disturbances were detected at the follow-up period and higher levels could be found in the recipients with infectious diseases. Triglyceride is usually degraded by lipoprotein lipase to produce free fatty acids, that may activate the nuclear factor-κB (NF-κB) role in the inflammatory response and resulting infectious diseases in kidney transplantation recipients (31, 32). Activated macrophages can inhibit lipoprotein lipase production to increase triglyceride levels by releasing TNF-α and IL-1 which may also be another factor that can promote the occurrence of infection (33).

A simple and novel nomogram consisting of 8 factors has been established in our study and it was also evaluated by ROC and benefit curves. Honestly, requirements for albumin and RBC infusions, the levels of triglyceride, uric acid, creatinine, globulin, neutrophil percentage, and WBC are not specific and the results of them are easily available in clinical practice. However, they can access the status of immunity and nutrition as well as the function of the allograft. Thus, the novel nomogram is worthy of clinical applications. Yet, limitations should still be mentioned. First, as the vast majority of infected kidney transplantation recipients have viral infections, the nomogram cannot be extrapolated to all range of infectious diseases. Second, invasive devices and catheter indwelling may disrupt the mucosal barrier and lead to the incidence of infectious diseases. However, some important confounding factors did not include in LASSO regression model of our study. Whether the recipients need mechanical ventilation or central venous catheter should be further analyzed in future research. Third, the type of infection is quite complex in adult kidney transplantation recipients which consists of simple and mixed infection. They were not further distinguished in our prediction model and studies with larger sample size are needed in the future. In addition, experimental validation of the nomogram through future studies is warranted.

## Conclusions

Adult kidney transplantation recipients are high-risk hosts for infectious diseases. The novel nomogram consisting of 8 factors reveals good predictive performance and may promote the reasonable antimicrobial prescription. Thus, it is possible to decrease the burden of health economics and contain the trends of antimicrobial resistance. More external validations are required to confirm its effectiveness for further clinical application.

## Data availability statement

The raw data supporting the conclusions of this article will be made available by the authors, without undue reservation.

## Ethics statement

The studies involving human participants were reviewed and approved by the Ethics Committee of Renji Hospital. Written informed consent for participation was not required for this study in accordance with the national legislation and the institutional requirements.

## Author contributions

Conception and design: Y-TY, CZ, and X-DY. Administrative support: MZ. Provision of study materials or patients: R-YC, S-YZ, D-WL, and Y-TY. Collection and assembly of data: SZ. Data analysis and interpretation: SZ, CZ, and Y-TY. Manuscript writing and final approval of manuscript: All authors.

## Funding

This work was supported by the Clinical Plus Excellence Program of Renji Hospital (No. 2021ZYA022); Shanghai Shenkang Hospital Development Center, Clinical Skills and Clinical Innovation Program (No. SHDC2020CR5012); Clinical Scientific Research Innovation Cultivation Program, Renji Hospital, School of Medicine, Shanghai Jiao Tong University (No. RJPY-D2X-010).

## Conflict of interest

The authors declare that the research was conducted in the absence of any commercial or financial relationships that could be construed as a potential conflict of interest.

## Publisher's note

All claims expressed in this article are solely those of the authors and do not necessarily represent those of their affiliated organizations, or those of the publisher, the editors and the reviewers. Any product that may be evaluated in this article, or claim that may be made by its manufacturer, is not guaranteed or endorsed by the publisher.
